# Treatment of Diethyl Phthalate Leached from Plastic Products in Municipal Solid Waste Using an Ozone-Based Advanced Oxidation Process

**DOI:** 10.3390/ma12244119

**Published:** 2019-12-09

**Authors:** Sankaralingam Mohan, Hadas Mamane, Dror Avisar, Igal Gozlan, Aviv Kaplan, Gokul Dayalan

**Affiliations:** 1 Environmental & Water Resources Engineering, Department of Civil Engineering, Indian Institute of Technology Madras, Chennai, Tamil Nadu 600036, India; smohan@iitm.ac.in (S.M.); gkld93@gmail.com (G.D.); 2 Environmental Engineering Program, School of Mechanical Engineering, Faculty of Engineering, Tel Aviv University, Tel Aviv 69978, Israel; hadasmg@tauex.tau.ac.il; 3 School of Earth Sciences, The Water Research Center, Hydrochemistry, Tel Aviv University, Tel Aviv 69978, Israel; gozlan.igal@gmail.com (I.G.); avivkaplan@tauex.tau.ac.il (A.K.)

**Keywords:** advanced oxidation, diethyl phthalate (DEP), leachate, peroxone, solid waste, plastic, landfill

## Abstract

Plastic products in municipal solid waste result in the extraction of phthalates in leachate that also contains large amounts of organic matter, such as humic substances, ammonia, metals, chlorinated organics, phenolic compounds, and pesticide residues. Phthalate esters are endocrine disruptors, categorized as a priority pollutant by the US Environmental Protection Agency (USEPA). Biological processes are inefficient at degrading phthalates due to their stability and toxic characteristics. In this study, the peroxone (ozone/hydrogen peroxide) process (O_3_/H_2_O_2_), an O_3_-based advanced oxidation process (AOP), was demonstrated for the removal of diethyl phthalate (DEP) in synthetic leachate simulating solid-waste leachate from an open dump. The impact of the O_3_ dose during DEP degradation; the formation of ozonation intermediate by-products; and the effects of H_2_O_2_ dose, pH, and ultraviolet absorbance at 254 nm (UVC) were determined during ozonation. Removal of 99.9% of an initial 20 mg/L DEP was obtained via 120 min of ozonation (transferred O_3_ dose = 4971 mg/L) with 40 mg/L H_2_O_2_ in a semi-batch O_3_ system. Degradation mechanisms of DEP along with its intermediate products were also determined for the AOP treatment. Indirect OH radical exposure was determined by using a radical probe compound (pCBA) in the O_3_ treatment.

## 1. Introduction

Rapid urbanization and rising living standards have a major influence on the quantity and characteristics of municipal solid waste (MSW), further complicating the management and maintenance of sanitary landfills. Leachate is generated by the interaction of MSW with water that percolates through the landfill, producing highly polluted wastewater [1]. Many factors govern the quality and quantity of leachate, such as the composition of the waste, piling, seasonal variation, landfill technique, and structure of the landfill. In developed countries, possible options for leachate treatment include co-treatment with municipal wastewater and transportation to an ex situ treatment facility; however, even these treatment processes are not practiced nor affordable. Other advanced techniques include reverse osmosis and recirculation of the concentrated leachate back to the landfill to avoid groundwater contamination [2]. In many developing countries, open dumpsites serve as a final disposal point for the MSW; these are a burden for the environment as the leachate is not collected or treated. One of the major concerns in the generation of leachate, in terms of both quantity and quality, is groundwater contamination [1]. Leachate extracts multiple contaminants from MSW and creates complex interactions between hydrological and biogeochemical reactions. Leachate contains a large amount of organic matter consisting of humic substances, along with ammonia nitrogen, heavy metals, chlorinated organics, and phenolic compounds.

Phthalate esters, known as phthalates, are plasticizers that are used to increase the quality and durability of polymers [3]. Phthalates leach and migrate from the plastic products into the environment as they are bonded to the products by physical and not chemical means, posing serious environmental and human health problems [4]. Phthalates, considered priority pollutants, have broad applications worldwide; they have a high biological toxicity to humans and are considered endocrine disruptor compounds (EDC) by the US Environmental Protection Agency (USEPA), European Environmental Agency (EEA), and other environmental agencies [5]. Specifically, diethyl phthalate (DEP) is commonly found in leachate and groundwater due to its high solubility and wide use in many items, among them being cosmetics, paints, and toys [5]. The USEPA-issued national primary drinking water regulation regarding organic chemical contaminants for DEP is 0.006 mg/L, which is the maximum permissible limit.

Studies have demonstrated the removal of organic contaminants and heavy metals from landfill leachate by various technologies, including pre-treatment techniques, such as a coagulation–flocculation process, stripping, and precipitation methods [6], followed by discharge to the municipal wastewater treatment plant, adsorption, or oxidation. Advanced oxidation processes (AOPs) have been successfully implemented for the degradation of recalcitrant substances from stabilized leachate (i.e., old leachate, which is less biodegradable) and to improve its biodegradation by increasing the ratio of biochemical to chemical oxygen demand (BOD_5_/COD) [7].

Ozone (O_3_) alone and in combination with different processes has been shown to efficiently degrade bulk organic contaminants from wastewater [8]. Organic compounds in leachate can be oxidized by either a direct reaction with O_3_, or by O_3_ decomposition to form the non-selective hydroxyl (OH) radicals at an alkaline pH; alternative methods include the addition of an oxidant such as hydrogen peroxide (H_2_O_2_) (termed peroxone process) or the addition of a catalyst at the natural water pH (7–8) [9]. For example, Amr et al. studied the removal of COD and color from a municipal landfill leachate using O_3_ combined with a zinc sulfate oxidation process and obtained 90% and 99% removal for COD and color, respectively, with a 1 g/6 g ZnSO_4_ dosage (COD_0_/Zn) at 180 min of treatment time [10]. In addition, Amr et al. compared and optimized COD and color removal for a stabilized landfill leachate using three different AOP systems: O_3_ alone, O_3_/fenton, and O_3_/persulfate [11]. The latter showed an improved performance efficiency compared to the other treatment processes for the removal of color, ammonia, and COD. Asaithambi et al. showed that the synergistic effect of a O_3_/sonication/fenton-based AOP was more effective in treating leachate with minimal electrical energy for high removal efficiency of 100% color and 95% COD [4]. Several studies have shown the removal of DEP in water (not in leachate) using an AOP for the generation of OH radicals, such as ultraviolet radiation (UV)/TiO_2_ [12], O_3_ [13] and UV/H_2_O_2_ [5]. These studies demonstrated that OH radicals play a dominant role in AOP efficacy for DEP removal in water.

Due to the high percentage of plastics in MSW composition, there is a high concentration of phthalates in the leachate. However, the removal of phthalates by O_3_-based AOP from the complex matrix of MSW leachate has never been explored. In this study, an O_3_-based AOP—the peroxone process (O_3_/H_2_O_2_)—was tested for the removal of DEP in a synthetic leachate simulating an MSW leachate from an open dump. The specific goal of this study was to demonstrate the impact of O_3_ and H_2_O_2_ dose during degradation on DEP removal from the leachate and the formation of ozonation intermediate by-products, in addition to changes in COD, pH, and UVC during the process.

## 2. Materials and Methods

### 2.1. Chemical Reagents and Leachate Analysis

Leachate was prepared using DEP (>98%), phthalic acid (>98%), phthalic anhydride (>99%), 4-hydroxy phthalic acid (>98%) (Sigma Aldrich, USA), potassium hydrogen phthalate (KHP), glucose (C_6_H_12_O_6_), ammonium sulfate ((NH_4_)_2_SO_4_), ammonium chloride (NH_4_Cl), copper sulfate (CuSO_4_), lead nitrate (Pb(NO_3_)_2_), nickel sulfate (NiSO_4_), potassium dichromate (K_2_Cr_2_O_7_), manganese sulfate (MnSO_4_), zinc sulfate (ZnSO_4_), propionic acid (C_3_H_6_O_2_), pentanoic acid (C_5_H_10_O_2_), and hexanoic acid (C_6_H_12_O_2_) (Holland Moran, Israel). Analytical grade H_2_O_2_ (30% *w*/*w*) was obtained from Merck Chemicals (USA). Stock solutions were prepared by dissolving each compound in deionized water (Direct-Q3 UV System, Millipore, France). COD test kits with a measuring range of 0 to 15,000 mg/L O_2_ were purchased from Lavibond (England), and were based on the dichromate method and determination in a Hach spectrophotometer. The UV absorbance coefficients of the samples with different H_2_O_2_ concentrations were measured using a UV-visible spectrophotometer (Varian, Cary 100BIO, Australia). Spectra were collected in quartz cuvettes using a wavelength range of 200–800 nm.

### 2.2. Synthetic Leachate Preparation

The composition of the synthetic leachate was adapted from a previous study [14] and modified based on analyzed leachate samples from the Perungudi open dumpsite, Chennai, India, and previous data in the literature [15] (Table 1). The leachate characteristics used in this study simulated numerous samples of municipal solid waste, open dumpsite leachate from Chennai, India. In this leachate, diethyl phthalate (DEP) was detected at an average concentration of 17.2 mg/L in the open dumpsite. The leachate used in this study simulates young leachate.

### 2.3. Experimental Setup and Procedure

**Figure 1:**
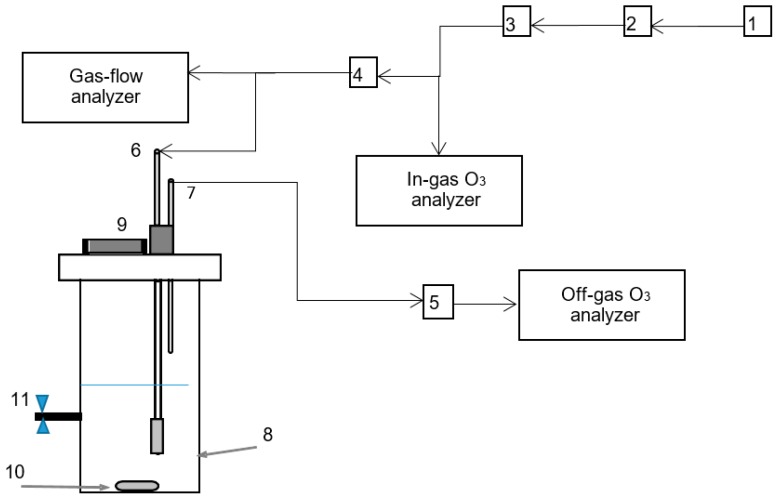
Semi-batch O_3_ system experimental setup: (1) O_2_ gas and flow-rate gauge; (2) air drier and humidity indicator; (3) O_3_ generator; (4) gas inlet gauge; (5) air drier for O_3_-saturated air; (6,7) gas inlet and outlet, respectively; (8) O_3_ diffuser; (9) solution inlet; (10) magnetic stir bar; and (11) sampling port.

O_3_ experiments were performed in semi-continuous batch reactors that allowed for the continuous addition of O_3_ to a fixed batch of leachate with added DEP, as shown in Figure 1. O_3_ gas was generated using an O_2_-fed O_3_ generator (up to 4 g/h; BMT 802N, Germany) and the O_2_–O_3_ gas mixture was bubbled directly into a 100-mL glass reactor (Figure 1). The reactor was 14 cm in height and 3.5 cm in diameter, with a diffuser size of 2.35 cm^3^ and a nominal pore size of 25 µm, as described in a previous study [16]. An O_3_-flow control valve was used to determine the O_3_ flow, which was maintained at 0.2 L/min operated under ambient conditions.

The transferred O_3_ dose (TOD) is an empirical parameter for determining the accumulated amount of O_3_ transferred to the water that reacts with the examined material. Although this parameter is usually used for the prediction of micropollutant elimination during the ozonation of municipal wastewater effluents, it can also serve for the determination of O_3_ accumulation in leachate. Here, the O_3_ dose was estimated using continuous measurements of the O_3_ concentration in the gas phase at the inlet and outlet (off gas) of the reactor:

(1) $$\mathrm{Transferred}\;\mathrm{Ozone}\;\mathrm{Dose}\left(\frac{{\mathrm{mgO}}_{3}}{L}\right)=\frac{\sum \{{\left(C_{O_{3},\mathrm{in}}-C_{O_{3},\mathrm{out}}\right)}_{mg/L}\cdot {\mathrm{gas}\;\mathrm{flowrate}}_{L/min}\}\times dt_{min}}{{\mathrm{volume}}_{L}}$$

where *C*_O_3_,in_ is the O_3_ concentration in the inlet stream, *C*_O_3_,out_ is the O_3_ concentration in the outlet stream, representing the unreacted O_3_ exiting the reactor, and *dt* is the time interval between measurements, set at 1 min.

DEP (20 mg/L) was spiked into the synthetic leachate with the addition of 0, 5, 10, 20, 30, 40, and 50 mg/L of H_2_O_2_ to study the impact of H_2_O_2_ on DEP degradation along with the generated intermediate transformation products. The parameters recorded with the O_3_ and O_3_/H_2_O_2_ processes were COD, UVC, and pH. The impact of OH radicals on DEP-degradation kinetics using direct ozonation was determined in the presence of the radical scavenger tert-butanol (t-BuOH) (50, 100, 150, 200 mM). The radical probe compound para-chlorobenzoic acid (pCBA) was used at concentrations of 10 mg/L and 20 mg/L to determine the steady-state OH radical concentration for the degradation of DEP.

### 2.4. High-Performance Liquid Chromatography (HPLC) Analysis

An Agilent 1100 series HPLC system with single-wavelength UV detector was used for the analysis of both DEP and pCBA. The column was a Kinetex C18-XB column, 100 × 3.0 mm, 2.6 µm at 40 °C, with a Gemini C18 4 × 2.0 mm pre-column guard. The pump was set to an isocratic program of 40% water, 60% MeOH, and 0.1% formic acid (*v*/*v*), at a flow rate of 0.5 mL/min. The injection volume was 30 µL and the detector wavelength was set to 228 nm for DEP (retention time 3.0 min) and 243 nm for pCBA (retention time 2.5 min).

### 2.5. LC-MS Analysis

LC-MS analysis of the DEP degradation products phthalic acid and p-hydroxy phthalic acid (obtained after sample treatment with O_3_/H_2_O_2_) was performed using HPLC (Agilent 1100) coupled to MS (Q-Tof, Waters, model Premier) via an electrospray ionization (ESI) interface in positive mode, using the Kinetex C18-XB column. The column temperature was set to 40 °C, the flow rate to 0.5 mL/min, and the injection volume was 30 µL. The HPLC mobile phase consisted of water (A) and methanol (B) with 0.1% formic acid. The elution gradient was initiated with 20% B, held for 2 min, increased to 65% over 11 min, and then held at 65% for 5 min.

## 3. Results and Discussion

### 3.1. DEP Removal in O_3_ and O_3_/H_2_O_2_ Processes

**Figure 2:**
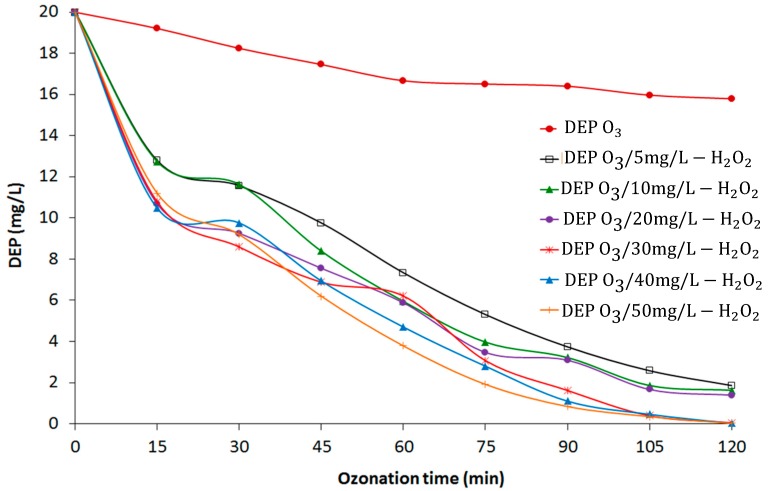
Diethyl phthalate (DEP) removal with peroxone process.

Synthetic leachate was spiked with 0.5 M (20 mg/L) DEP at pH 7.5 with various H_2_O_2_ concentrations (0, 5, 10, 20, 30, 40, and 50 mg/L) and ozonated to determine the ozonation kinetics. Figure 2 shows the degradation of DEP with the O_3_ and O_3_/H_2_O_2_ processes. The DEP concentration decreased by 21% with 120 min of ozonation only (TOD = 4971 mg/L). To increase the rate of DEP degradation, H_2_O_2_ was added to accelerate the production of OH radicals for the efficient degradation of DEP from leachate using the peroxone process.

The degradation rate of DEP increased with increasing H_2_O_2_ concentration. The addition of 0 to 50 mg/L H_2_O_2_ increased the degradation rate from 21% to 99.9% at 120 min of ozonation. The reaction process can be explained by the following equations, where in Equation (2), the conjugate base of the H_2_O_2_ concentration is pH dependent.

(2) $${\mathrm{OH}}^{-}+O_{3}\to O_{2}+{\mathrm{HO}}_{2}^{-}\overset{H+}{↔}H_{2}O_{2}$$

(3) $${\mathrm{HO}}_{2}^{-}+O_{3}\to {\mathrm{HO}}_{2}^{\cdot }+O_{3}^{\cdot -}$$

(4) $${\mathrm{HO}}_{2}^{\cdot }↔H^{+}+O_{2}^{\cdot -}$$

(5) $$O_{2}^{\cdot -}+O_{3}\to O_{2}+O_{3}^{\cdot -}$$

(6) $$O_{3}^{\cdot -}+H^{+}\to •\mathrm{OH}\left(\mathrm{hydroxy}\right)+O_{2}$$

(7) $$•\mathrm{OH}+O_{3}\to {\mathrm{HO}}_{2}^{\cdot }\left(\mathrm{proxy}\right)+O_{2}$$

(8) $$\mathrm{DEP}+O_{3}\to \mathrm{Products}+\mathrm{Intermediate}\;\mathrm{compounds}$$

(9) DEP+•OH→Products+Intermediate compounds

•OH + Intermediate products → CO_2_ + H_2_O (10)

The addition of H_2_O_2_ to O_3_ results in O_3_ decomposition and OH radical (•OH) formation. At high pH, the concentration of HO_2_^−^ increases and hence the concentration of •OH increases [13]. At an acidic pH, ozone reacts selectively and slowly with organics, whereas with increasing pH ozone decomposition is accelerated and radical reactions occur via the reaction of ozone with some compounds known as initiators, such as hydrogen peroxide (H_2_O_2_) [16]. Reactions for O_3_/H_2_O_2_ are similar to those for O_3_ alone with added H_2_O_2_ dosing the system. The peroxone reaction is:

(11) $$H_{2}O_{2}+H_{2}O↔{\mathrm{HO}}_{2-}+H_{3}O+O_{3}+{\mathrm{HO}}_{2-}\to •\mathrm{OH}+O_{2-}+O_{2}.$$

An increase in DEP degradation with the addition of H_2_O_2_ to the ozonation process was due to the formation of OH radicals. According to Figure 2, the optimal H_2_O_2_ concentration was ≈40 mg/L with almost complete DEP removal (99.9%). No additional decrease in DEP concentration was obtained with the addition of 50 mg/L H_2_O_2_ to the peroxone treatment.

Supplementary Figure S2 shows the change in pH during the peroxone process. During the ozonation treatment, the pH gradually decreased from 7.5 to 5.1 with time and with the addition of H_2_O_2_. Alkaline pH enhances the formation of OH radicals as more hydroxide ions are present (Equations (12) and (13)). These hydroxide ions initiate O_3_ decay.

O_3_ + OH^−^ → HO_2_^−^ + O_2_ (12)

O_3_ + HO_2_^−^ → •OH + O_2_^−^ + O_2_ (13)

The produced •OH (Equation (13)) can subsequently introduce further chemical reactions with O_3_, resulting in an increased OH radical formation. In addition, the pH of the wastewater influences the acid/base equilibria of some compounds, as well as the O_3_ reaction rate.

### 3.2. Effect of O_3_/H_2_O_2_ Process on COD, UVC, and pH

**Figure 3:**
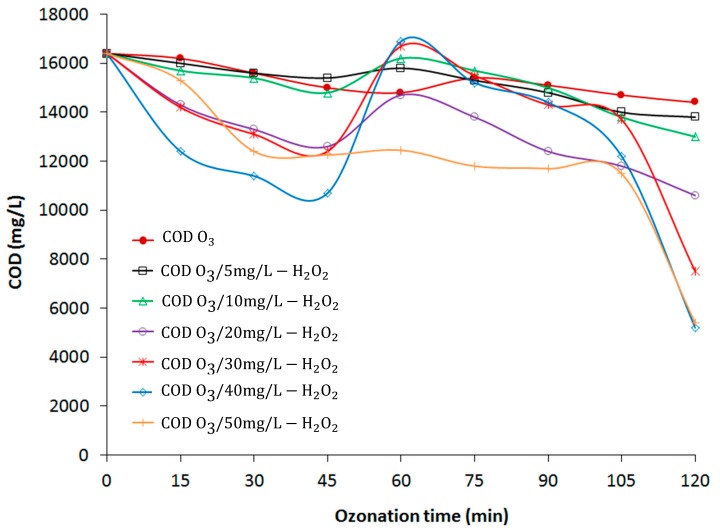
Influence of O_3_ and O_3_/H_2_O_2_ on chemical oxygen demand (COD) removal.

**Figure 4:**
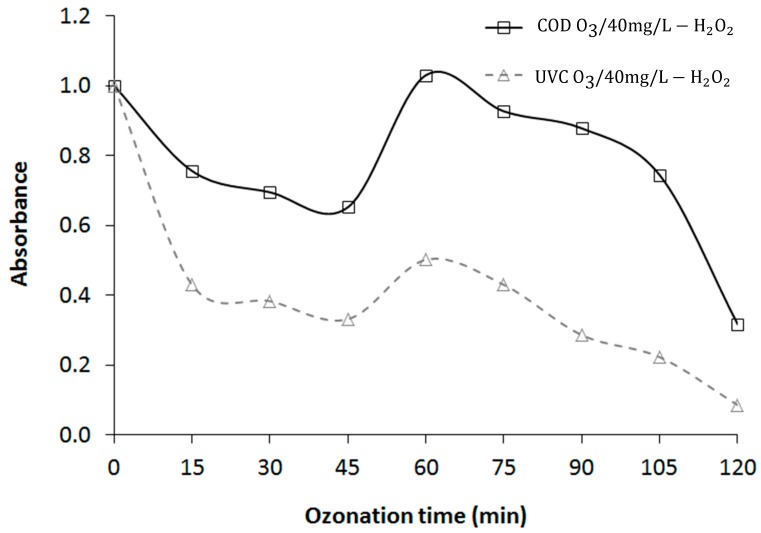
COD versus UVC for O_3_ with 40 mg/L H_2_O_2_.

Leachate is characterized by a high concentration of organic matter, as determined by COD value, which refers to the amount of specific oxidant reacting with the sample under controlled conditions [17]. In this study, the initial COD value in the leachate was ≈16,400 mg/L. The mixed solid wastes that were deposited in the open dump in a non-segregating method produced a high COD and probably required a high concentration of oxidant to degrade the complex compounds and increase the leachate biodegradability. Figure 3 and Figure 4 show the effects of O_3_ and O_3_/H_2_O_2_ on COD and the correlation between COD and UVC, respectively. Figure 3 illustrates that only 12.2% of the COD was removed, from 16,400 mg/L to 14,400 mg/L, during 120 min of O_3_ treatment. The presence of H_2_O_2_ (5, 10, 20, 30, 40, and 50 mg/L) resulted in increased COD removal, from 15.9% (O_3_/5 mg/L H_2_O_2_) to 68.9% (O_3_/50 mg/L H_2_O_2_) for a final COD concentration after 120 min of ≈5800 mg/L. Removal of COD indicates that the addition of H_2_O_2_ increased the formation of OH radicals, which in turn mineralized the organic compounds. Tizaoui et al. reported an improvement in COD removal from 27% to 48% using semi-batch O_3_ and O_3_/2000 mg/L H_2_O_2_, respectively [18], which was a considerably higher amount of H_2_O_2_ than that used in the present study.

Figure 3 shows that the COD decreased gradually during the initial O_3_ and O_3_/H_2_O_2_ processes due to the availability of OH radicals. Further, an increase in COD concentration was observed at 60 min (except for the O_3_ treatment alone), indicating the formation of organic acids during DEP degradation by AOP [13]. As the peroxone treatment continued, COD decreased further due to additional oxidation of the intermediates by OH radicals to the final COD. The COD removal with 40 mg/L H_2_O_2_ was similar to that with 50 mg/L H_2_O_2_, showing that an increased H_2_O_2_ concentration does not always result in increased COD-removal rates. The increase in H_2_O_2_ concentration leads to a change in its role from OH radical producer to inhibitor of O_3_ decomposition by free radicals [19].

(14) $$O_{3}+{\mathrm{HOO}}^{-}\to O_{3}{}^{\cdot -}+{\mathrm{HO}}_{2}{}^{\cdot }$$

According to Peretz et al. [16], increasing the concentration of H_2_O_2_ can cause further decomposition of ozone while yielding the ozonide radical anion O_3_, which could be detected as dissolved ozone. This may result in a pseudo increase in the dissolved ozone concentration. Similarly, UVC at 254 nm followed the COD removal trend, probably due to its high concentration, for an optimum dosage of O_3_/40 mg/L H_2_O_2_ (Figure 4).

Numerous studies have shown that UVC can be used to probe the micropollutant removal in secondary wastewater effluent, e.g., to track carbamazepine in a secondary effluent [20]. However, the current study showed that in the complex leachate matrix, it was not possible to track the degradation of the target pollutant using UVC. Leachate has many complex organic substances and heavy metals that interfere with pollutant removal, and Figure 4 proves that the UVC reflected the degradation of COD in leachate, indicating that it cannot be used as a tracking model for DEP removal.

### 3.3. Effect of t-BuOH in the Kinetic Evaluation

**Figure 5:**
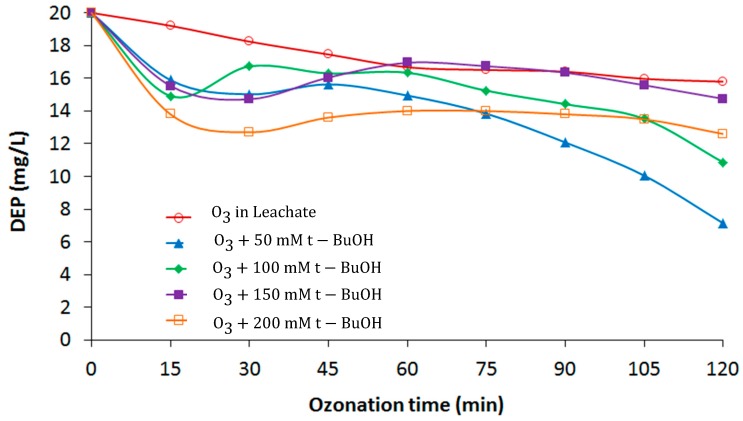
Diethyl phthalate (DEP) removal in leachate for different tert-butanol (t-BuOH) concentrations.

The radical scavenger t-BuOH was added to quench the formation of OH radicals during the ozonation process because the rate constant of O_3_ for DEP is very low (K_O_3__ = 0.06–0.1 M^−1^s^−1^) and maximum DEP removal was achieved using the OH radicals. To determine the impact of direct ozonation on DEP degradation, t-BuOH was added to the leachate at 50 mM, 100 mM, 150 mM, and 200 mM. Figure 5 shows the removal of DEP in leachate with different concentrations of t-BuOH compared to the direct ozonation of the leachate. Ozonation alone yielded ≈21% DEP removal from the leachate (120 min; TOD = 4971 mg/L), whereas addition of t-BuOH to the leachate increased the removal rate of DEP instead of decreasing it.

From Figure 5, the addition of 50 mM t-BuOH gave 64.3% DEP removal; increasing the t-BuOH concentration to 150 mM resulted in less DEP removal (≈26.3%), and 200 mM t-BuOH then increased the DEP degradation to a final concentration of 12.6 mg/L after 120 min. Thus, even after the addition of the OH radical scavenger, there was more degradation of DEP than with ozonation alone. The addition of t-BuOH surprisingly did not suppress the OH radical formation. Instead, the addition of t-BuOH enhanced the DEP removal in comparison to the ozone treatment without the added t-BuOH. t-BuOH may contribute to the formation of transesterification products, which in turn may enhance the OH radical’s formation [21] and the degradation of DEP. Transesterification is the process of exchanging the organic group of an ester with the organic group of an alcohol. Consequently, in the leachate matrix, t-BuOH cannot be used to evaluate the impact of direct ozonation on DEP degradation.

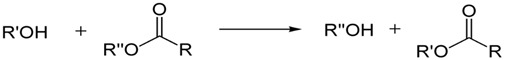
(15)

### 3.4. OH Radical Exposure

O_3_ exposure $\int \left[O_{3}\right]dt$ and OH radical exposure ∫[·OH]dt, under the R_ct_ concept, were monitored during ozonation by the depletion rate of an in situ OH radical probe compound (pCBA). R_ct_ represents the ratio of the OH radical concentration to the O_3_ concentration in a given body of water/wastewater (R_ct_ = [•OH]/[O_3_]) [22]. Degradation of pCBA in leachate can be predicted using the O_3_ kinetics and R_ct_. The OH radical and O_3_ exposures in a steady state are determined specifically for every application since they are directly affected by water-quality parameters:

(16) $$\frac{c}{c_{0}}=exp\left(-k_{O_{3}}\int \left[O_{3}\right]dt-k_{\cdot \mathrm{OH}}\int \left[\cdot \mathrm{OH}\right]dt\right),$$

where *k*_O_3__ represents the rate constant of O_3_ and *k*_OH_ represents the rate constant of OH radicals for the O_3_ probe compound pCBA. The direct reaction of the resistant tracer with O_3_ is negligible; hence its contribution to compound removal can be eliminated from the equation, according to Equation (15). The “OH-radical probe method” involves an indirect determination of oxidant exposures, where the OH radical exposure is back-calculated from the removal of the O_3_-resistant probe compound (pCBA) [22,23,24,25]:

(17) $$\frac{c}{c_{0}}=exp\left(-k_{\cdot \mathrm{OH}}\int \left[\cdot \mathrm{OH}\right]dt\right)$$

However, an accurate O_3_-exposure measurement in wastewater effluent is problematic since the rapid reaction of effluent organic matter and even effluent particles [20] with O_3_ results in rapid O_3_ depletion in the first milliseconds of the reaction [23]. An alternative concept, referred to here as the “O_3_ probe method,” suggests that O_3_ exposure can be back-calculated from the removal of an internal tracer (a moderately or rapidly reacting probe compound) that reacts with both O_3_ and OH radicals following the ozonation of wastewater effluents. DEP cannot be used as the internal probe compound for OH radical estimation due its degradation to both superoxide and peroxy radicals [26].

**Figure 6:**
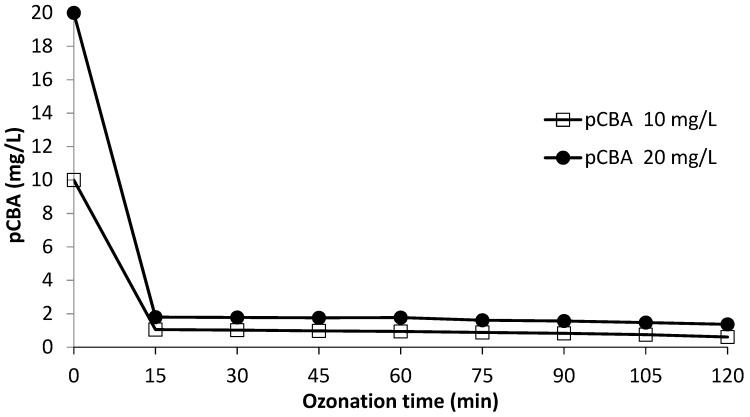
Para-chlorobenzoic acid (pCBA) degradation for a semi-batch O_3_ process.

Using the R_ct_ concept, the OH radical probe compound pCBA was spiked into the leachate (at 10 mg/L and 20 mg/L) containing 20 mg/L DEP, and its final concentration was determined after ozonation for different time intervals (Figure 6). Table 2 shows the OH exposure for 10 and 20 mg/L pCBA, respectively; with K_O_3__ and K_OH_ values for the O_3_ treatment of 0.15 M^−1^s^−1^ and 5 × 10^9^ M^−1^s^−1^.

### 3.5. Formation of Intermediate Transformation Products

**Figure 7:**
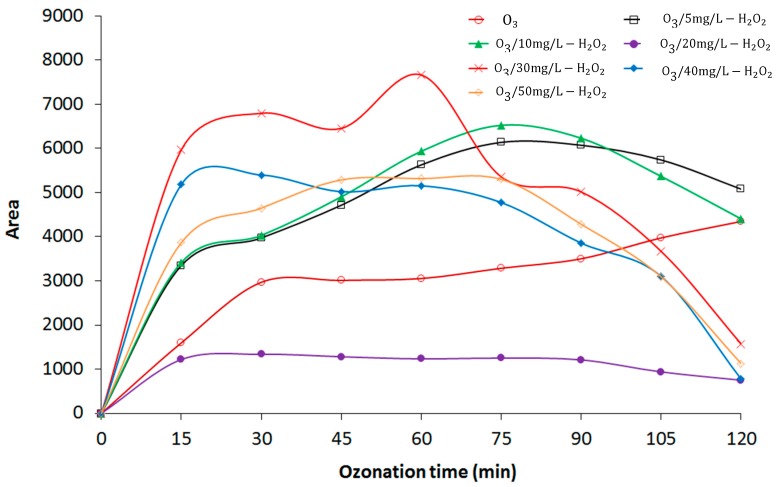
Phthalic acid as an intermediate diethyl phthalate (DEP) degradation product.

**Figure 8:**
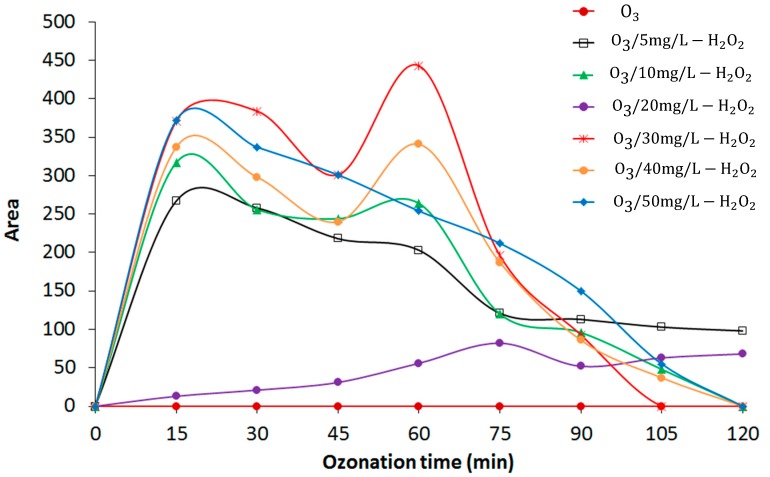
4-Hydroxy phthalic acid as an intermediate diethyl phthalate (DEP) degradation product.

DEP degradation contributes to the formation of various byproducts during the O_3_ and O_3_/H_2_O_2_ treatment processes, along with the production of organic acids, such as succinic acid, malonic acid, and glutaric acid [13], as well as three possible intermediate products: phthalic acid, 4-hydroxy phthalic acid, and phthalic anhydride [13]. Figure 7 and Figure 8 show the trend analysis of the areas for phthalic acid and 4-hydroxy phthalic acid, respectively. Intermediate product formation occurs upon DEP degradation due to cleavage of its chain and hydroxylation of the aromatic ring.

Previous studies have shown that during ozonation [10], DEP does not readily react with O_3_ molecules, indicating a very low reaction-rate constant. Consequently, O_3_ decay via DEP degradation results in the formation of intermediate DEP-oxidation products. During the 120-min O_3_ treatment, there was a consistent increment of phthalic acid (Figure 7), while 4-hydroxy phthalic acid was not produced using O_3_ alone (Figure 8), possibly due to a low concentration or absence of OH radicals that are expected to attack the aromatic ring at the para position.

In addition, Figure 7 and Figure 8 show that the areas of both phthalic acid and 4-hydroxy phthalic acid increased during the first 60 min of the O_3_/H_2_O_2_ treatment. As the treatment time increased, the intermediate products increased during the DEP degradation, reaching a maximum area, and then began to decrease as the experiment proceeded. Figure 7 shows that the area of phthalic acid decreased after 120 min while there was still residual phthalic acid in the leachate. 4-Hydroxy phthalic acid showed the same trend and there was a complete elimination of 4-hydroxy phthalic acid, except with 5 and 20 mg/L H_2_O_2_ after 120 min (Figure 8). Table 3 shows the second-order reaction rate constants for the O_3_ and OH radical with DEP.

## 4. Conclusions

The degradation of DEP by O_3_ alone and peroxone with varying concentrations of H_2_O_2_ was demonstrated. The simulated leachate had a high concentration of COD (16,400 mg/L) along with heavy metals, resulting in a complex matrix for the treatment process. DEP was not degraded by O_3_ alone [27], but it was degraded by the peroxone process due to the high rate removal by OH radicals (3.9 × 10^9^ M^−1^s^−1^). The COD removal of DEP by O_3_ with 40 mg/L H_2_O_2_ showed a 65% decrease in 120 min from the initial COD. Measurement of the in-gas and off-gas O_3_ dose during the treatment process revealed a rapid kinetic change in the initial treatment time that decreased toward the end of the treatment and influenced the oxidation process. The ozonation of leachate resulted in a minor change in pH due to the buffering effect of the bicarbonates. The OH radical exposure in a steady state was estimated using a radical probe compound, which confirmed the OH radical’s reaction with the leachate and its effect on the contaminant degradation. Two intermediate products were detected using HPLC–MS during the DEP degradation: phthalic acid and 4-hydroxy phthalic acid. They were confirmed using available reference compounds and ESI (+) MS spectra. A trend analysis was performed to estimate their area and degradation during the treatment process.

## Figures and Tables

**Table 1:** Synthetic leachate characteristics.

Parameter	Model Compound	Measured Value
pH	-	7.5
Chemical oxygen demand	KHP	16,400 mg/L
Phthalate	Diethyl phthalate	20 mg/L
Chloride	NH_4_Cl	1500 mg/L
Manganese	MnSO_4_	16 mg/L
Zinc	ZnSO_4_	12 mg/L
Lead	Pb(NO_3_)_2_	2 mg/L
Chromium	K_2_Cr_2_O_7_	1.5 mg/L
Copper	CuSO_4_	2.5 mg/L
Nickel	NiSO_4_	4.5 mg/L
Acetic acid	Organic acid	7 mg/L
Propionic acid	Organic acid	5 mg/L
Pentanoic acid	Organic acid	1 mg/L
Hexanoic acid	Organic acid	1 mg/L

**Table 2:** OH radical exposure for O_3_ treatment.

Ozonation Time (min)	pCBA (10 mg/L)	OH Exposure	pCBA (20 mg/L)	OH Exposure
0	10	0	20	0
15	1.06	4.50 × 10^−10^	1.8	4.82 × 10^−10^
30	1.02	4.57 × 10^−10^	1.78	4.84 × 10^−10^
45	0.98	4.65 × 10^−10^	1.76	4.86 × 10^−10^
60	0.94	4.73 × 10^−10^	1.77	4.85 × 10^−10^
75	0.88	4.86 × 10^−10^	1.61	5.04 × 10^−10^
90	0.83	4.98 × 10^−10^	1.57	5.10 × 10^−10^
105	0.75	5.18 × 10^−10^	1.48	5.21 × 10^−10^
120	0.61	5.60 × 10^−10^	1.37	5.37 × 10^−10^

pCBA: para-chlorobenzoic acid.

**Table 3:** Second-order rate constants for the reaction of O_3_ and OH radicals with diethyl phthalate.

Compound Name	Structure	K_O_3__ (M^−1^s^−1^)	K_OH_ (M^−1^s^−1^)
Diethyl phthalate	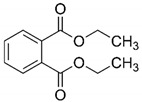	0.06–0.1 (Wen et al., 2011) 0.14	3–5 × 10^9^ (Haag et al., 2005)

## Supplementary Materials

The following are available online at https://www.mdpi.com/1996-1944/12/24/4119/s1, Figure S1. Transferred ozone dosage; Figure S2. Influence of O_3_ and O_3_/H_2_O_2_ on pH.
