# Supplementary material for: Treatment of Diethyl Phthalate Leached from Plastic Products in Municipal Solid Waste Using an Ozone-Based Advanced Oxidation Process
Source: Materials (Basel). 2019 Dec 9;12(24):4119. doi: 10.3390/ma12244119 (PMC6947371; doi:10.3390/ma12244119)
Supplement: Supplementary file 1 [file materials-12-04119-s001.pdf]

materials

Supplementary Materials: Treatment of Diethyl Phthalate Leached from Plastic Products in Municipal Solid Waste by Ozone-Based Advanced Oxidation Process

Mohan Sankaralingam [1], Hadas Mamane [2], Dror Avisar [3,*], Igal Gozlan [3], Aviv Kaplan [3] and Gokul Dayalan [1]

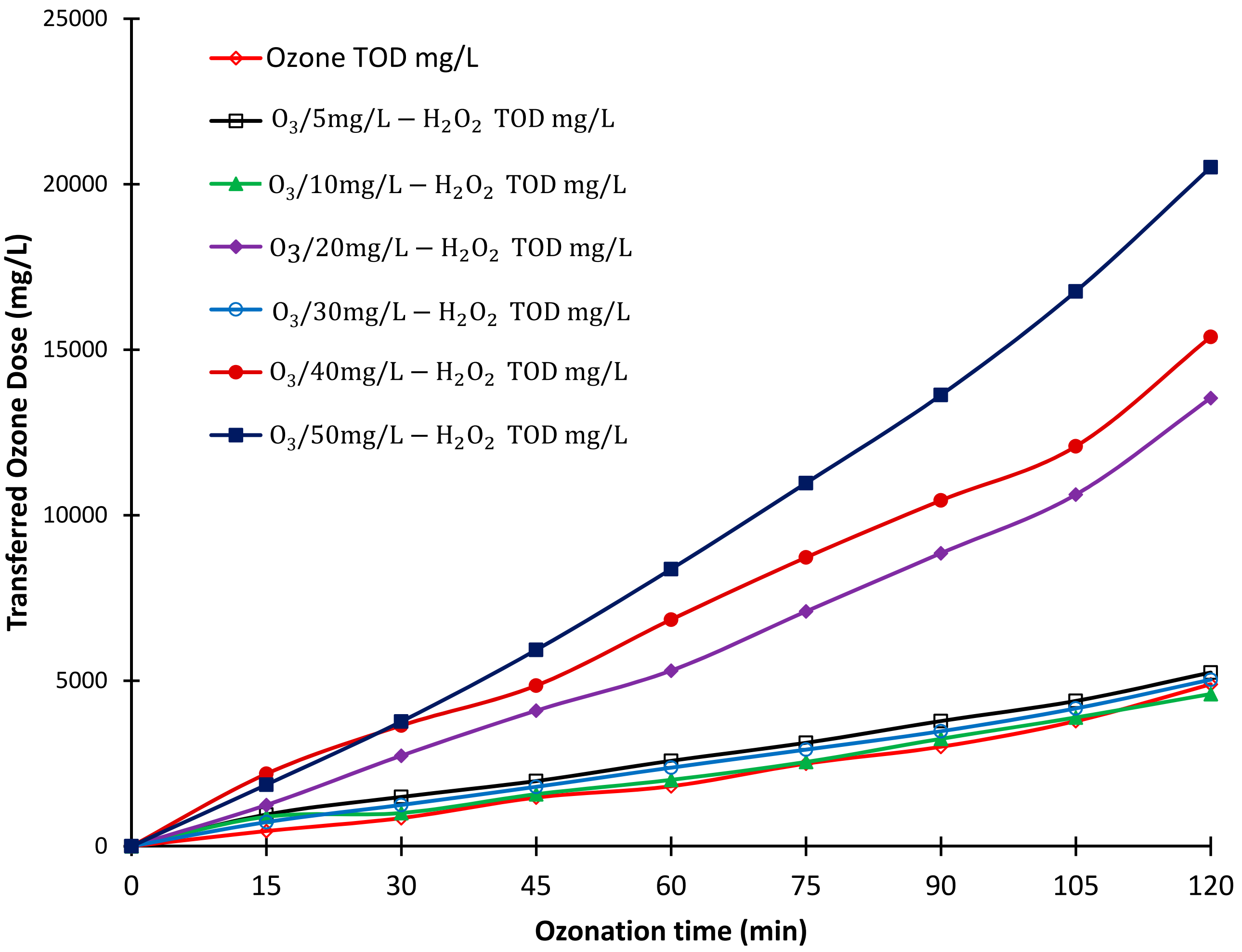

Figure S1. Transferred Ozone dosage.

materials

MDPI

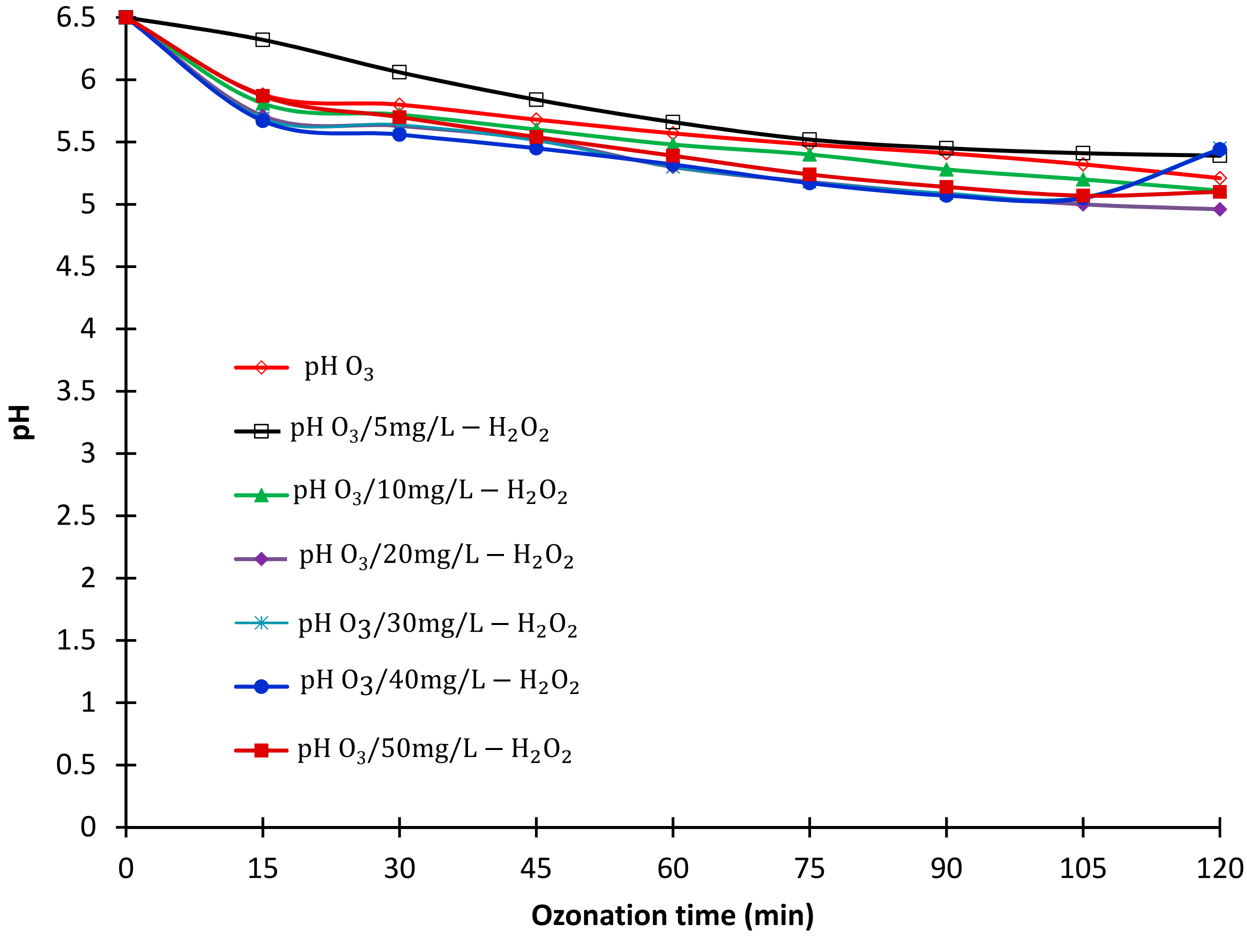

Figure S2. Influence of O_3 and O_3/H_2O_2 on pH.
